# A Novel Square-Stepping Exercise Program for Older Adults (StepIt): Rationale and Implications for Falls Prevention

**DOI:** 10.3389/fmed.2019.00318

**Published:** 2020-01-14

**Authors:** Eleftheria Giannouli, Tobias Morat, Wiebren Zijlstra

**Affiliations:** Institute of Movement and Sport Gerontology, German Sport University Cologne, Cologne, Germany

**Keywords:** balance, gait, mind-motor, dual-task training, aging, rhythmic auditory cueing, rhythmic auditory stimulation (RAS), variable practice

## Abstract

The ability to effectively execute compensatory steps is critical for preventing accidental falls, and consequently stepping training is an essential ingredient of fall prevention programs. In this paper, we propose a concept for stepping training that aims to maximize training effects by taking into account recent research evidence and a precise dosing of training ingredients. The concept addresses motor as well as cognitive falls-related aspects, it is suitable for individual as well as group based training, and it does not require costly equipment. Theory and evidence behind all of the training principles is reviewed, and an example of an exercise protocol is described in detail. Participants are presented with stepping patterns which they have to memorize and implement on a mat. In order to enable investigation of dose-response effects, the difficulty level systematically and gradually increases session by session based on four principles: execution speed, pattern complexity, pattern length and execution in dual-/multi-tasking conditions. The presented concept can be used as a framework for the development of further prevention and/or rehabilitation stepping exercise programs. Further studies using this exercise regimen or modified versions of it are encouraged.

## Introduction

In order to release older adults and healthcare systems from the burden of accidental falls, it is important to timely detect fall risk factors and develop effective interventions for falls prevention. Some of the strongest predictors for falls are deficits in lower-limb strength, balance, and gait performance (1). Specifically, accidental falls in older adults can be frequently attributed to the inability to step precisely on the ground and to incorrect weight-transferring during everyday activities such as (single-task) walking (2, 3) or in situations that require a simultaneous performance of several tasks (4). This can be explained by the age-related changes in spatiotemporal characteristics of stepping for balance; older individuals tend to: take too short steps or steps in the wrong direction (5, 6), to collide one leg against the other during oblique steps (7, 8), have slower stepping reactions (9) and need more attentional resources while walking under dual-task conditions (10).

The most important component of falls prevention exercise programs is balance training (11) since good balance is crucial for maintaining postural equilibrium and thus for the avoidance of falls. Balance exercise programs often focus on standing balance tasks, where the center of mass has to be statically controlled over the base of support. However, these lack in ecological validity and are not specific enough to cause neuromuscular adaptations that are actually required in balance-threatening situations. As mentioned earlier, in real life, maintaining balance to avoid a trip or a slip requires fast (rather than static/slow) stepping movements as well as high foot placement accuracy in order to initiate a correct step or inhibit a wrong one to quickly avoid an obstacle or an unexpected perturbation. Therefore, effective falls prevention and rehabilitation exercise programs should focus on performing precise, rapid and well-directed steps.

Stepping training is a form of highly specific balance training for falls prevention which directly addresses stepping capacity—a commonly executed protective strategy for maintaining balance in the everyday environment (12) and also an important fall risk factor in older adults (13, 14). A recent systematic review and meta-analysis showed, indeed, that both reactive and volitional stepping interventions reduce falls among older adults by ~50% (15).

In addition to this, physical activity in general and even more so structured physical exercise has shown to improve cognitive fall risk factors (16). In fact, combined physical and cognitive training (such as a stepping training containing additional cognitive tasks), may lead to larger improvements in cognitive and physical outcomes compared to physical or cognitive training alone (17), possibly with greater impacts on daily functioning. Thus, targeting deficits in both mobility and cognition through dual- and/or multi-tasking exercise programs is likely to represent the most effective strategy to minimize cognitive and physical declines in healthy older adults and therefore reduce the risk of falls and cognitive impairment.

Finally, regardless of type of training (strength/balance/stepping training), difficulty level (i.e., including increasingly challenging exercises e.g., either by adding a cognitive element, having additional movements, or increasing speed) is crucial for the success of falls prevention programs. This has been shown by systematic reviews (17, 18) as well as by a recent study monitoring movement characteristics of stepping exergames (19).

In this paper we propose a concept for stepping training (StepIt) that aims to maximize training effects in stepping capacity by taking into account recent research evidence and a precise dosing of training ingredients. In the next sections, we first review the theory and evidence behind Rhythmic Auditory Stimulation (RAS) used as an overall tool for stepping interventions aiming to prevent falls. Afterwards, we elaborate on all defined key elements of the proposed training by explaining their (neural) mechanisms. Finally, an exemplary protocol of the StepIt exercise program which includes all the aforementioned principles is described in detail and based on that, recommendations are presented on how this exercise program can be adjusted to fit needs of different purposes and populations. The presented concept can be used as a framework for the development of further prevention and/or rehabilitation stepping exercise programs.

## Rhythmic Auditory Stimulation

At the StepIt exercise program participants are presented with stepping patterns which they have to memorize and then execute on a grid-like rubber mat using the method of rhythmic auditory cueing as execution is done at paces delivered by a metronome. Rhythmic Auditory Stimulation (RAS) is “a neurologic technique using the physiological effects of auditory rhythm on the motor system to improve rhythmical movements like for example gait” (20). To cue movements, metronomes and music (or a combination of them, i.e., metronome tone embedded into music) are the most frequently used rhythmic auditory stimuli tools. They have been used very effectively in the context of RAS-based motor rehabilitation programs for patients suffering from various movement disorders (21), mostly as a result of neurological diseases like stroke (22, 23), Parkinson's disease (24, 25) and multiple sclerosis (26). It has been consistently reported that stepping in time to a metronome can improve pathological gait in neurological patients e.g., in terms of increased walking speed (27), reduced step time asymmetry and step time variability (28) and increased stride length (29) as well as in healthy, but fall-prone, older adults (24), even after only one training session (30).

This profound effect of auditory rhythm to the motor system is due to the rich connectivity between these two systems (auditory and motor) across a variety of cortical, subcortical and spinal levels. In fact, the motor system is so sensitive to stimulation by the auditory system that even simply listening to an auditory rhythm engages motor areas in the brain (31). The auditory system detects and processes temporal information with high precision and speed (it is actually about 20–50 ms faster and more precise than the visual and tactile systems (32) and subsequently projects them into motor structures in the brain, creating entrainment between the rhythmic signal and the motor response (33). In physics, entrainment is defined as the process in which one system's motion or signal frequency entrains the frequency of another system. In clinical language, entrainment refers to the process where the brain's internal timekeeper adjusts to external timekeepers (music/metronome etc.) enabling enhanced motor control in terms of increased anticipatory mapping and scaling of optimal velocity and acceleration gait parameters across the fixed movement interval (34). This movement “reprogramming” results in higher gait speed as well as smoother and less variable movement and muscle activation (35).

Neuroimaging studies have described the neural basis for auditory-motor entrainment. The auditory system has fiber connections from spinal motor neurons and up until the brain stem, subcortical, and cortical levels (34). Brain areas involved in rhythm processing, timing and duration perception, are closely related to those which control movement, such as the premotor cortex, supplementary motor area (SMA), cerebellum and basal ganglia. The cerebellum, which is involved in sensorimotor coupling, may monitor rhythmic patterns and adjust behavior to changing tempos and therefore control rhythmic auditory-motor synchronization. The putamen, and the basal ganglia in general, are associated with rhythmic events and beat perception (36).

Overall, the process of continuous entrainment resulting from the attempt to synchronize the movements with the rhythm requires repeated “error corrections.” This process takes place throughout the StepIt exercise program and gradually improves with practice until automatization is reached (37). This beneficial entrainment effect of rhythmic auditory cueing has been suggested to involve a variety of mechanisms: (i) supplement of sensory deficits present in fall-prone persons, (ii) neurophysiological changes, (iii) enhancement of auditory imagery, (iv) reduction in musculoskeletal activation variability, and (v) reduction of cognitive-motor interference (38).

## Difficulty Level Principles

One of the basic theories of training is that in general a skill will improve if it is practiced. Practicing a motor task that is varied along some task dimension is referred to as variable practice and it is considered to be a method that enhances the ability to transfer the learned task to a novel variation of the task or to a new environment (39). This happens because, similar to the mechanism just described for the RAS technique, the continuous attempt to adjust to altered conditions creates a trial-and-error mechanism that ultimately maximizes retention (40).

**Figure 1:**
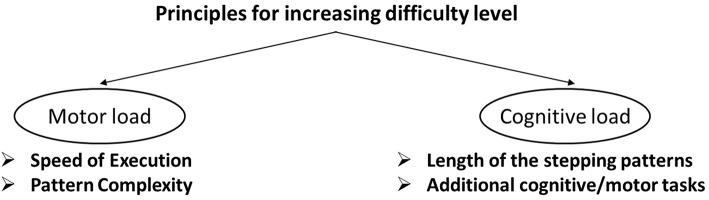
Principles for increasing difficulty level.

Progression during the StepIt exercise program is achieved by systematically manipulating four training principles, two addressing motor load and two addressing cognitive load. Motor load is increased gradually by increasing speed of execution and complexity of the stepping pattern. Cognitive load is increased gradually by extending the length of the stepping patterns and by adding additional cognitive/motor tasks (Figure 1). All four principles (execution speed, pattern complexity, pattern length and execution in [Dual-Task (DT)/Multi-Task (MT)], based on which progression was achieved, have been found to be important components of effective balance training/falls prevention exercise programs in former single studies (14, 19, 41).

### Execution Speed

In order to improve the ability to step quickly, the tempo given by the metronome on which participants have to execute the presented stepping patterns is increased across the training sessions. The ability to step quickly is a critical factor in avoiding a fall (42). Unfortunately, many community-dwelling older adults walk slower than the optimal speed for functioning optimally in the everyday life (e.g., to cross a street safely as a pedestrian).

Stepping exercise programs for fall-prone individuals who often show sensory, neuromuscular, and cardiopulmonary deficits may be enhanced by a variable practice schedule as manipulation of intensity by increasing movement speed increases demands in these systems which in turn may after repeated training result in an increase in physiologic reserve. Furthermore, stepping in high speeds transfers to a functional outcome as it simulates the actions that need to be taken in real life to avoid a fall. Indeed, high-intensity variable stepping training has shown to improve gait speed as well as other gait kinematics in frail older adults (43) as well as in neurological patients (44).

Regarding the neural mechanisms underlying the relevance of high speed training, studies suggest that plasticity associated with locomotor adaptation is speed-specific meaning that there are some neural networks for controlling locomotion that are recruited specifically for fast vs. slow walking (45). Recently, this is has been shown on a muscular level; different locomotor modules are recruited in different walking speeds to presumably meet additional functional demands with a speed increase (46). This separation in the control of fast and slow walking could possibly occur in the cerebellum, which is responsible for locomotor adaptation (45).

### Pattern Complexity

To avoid a trip or a slip in real life, besides speed, direction, and amplitude of the compensatory step is also crucial. Therefore, across sessions, complexity is manipulated by starting with patterns including only shorter, forward/lateral steps and progressing to wider, backward/oblique steps.

Stepping quickly in different directions and/or changing travel direction while walking, often referred to as steering (47) are two very important and also very complex aspects of balance and mobility of older adults. The majority of falls in old age happen while walking (48) due to a trip (40–60%) or a slip (10–15%) (49). Falls are 44% forward, 41% backward, and 33% concurrently or solely sideward (50). Studies have shown that in order to prevent falling, older adults, and especially fallers, prefer to modify their base of support (make a longer step to extend their margin of stability) by using stepping rather than feet-in-place strategies (grabbing nearby objects or rapidly extending the arms to prevent falling) (12, 51). These compensatory steps are often a response to both forward (82%) as well as sideward (70%) falls (50), indicating that the ability to take rapid steps in various directions could potentially prevent many falls. Studies have found that there is an age-related decrease of stepping speed in the forward, backward and sideward direction (52) and that this is more pronounced for fallers than non-fallers (53).

Most of the existing stepping exercise programs include forward and lateral steps. This is important as age-related deficits in lateral balance recovery are associated with fall incidence (8, 54) and most hip fractures occur after lateral falls (55), often because older adults tend to collide their legs against each other trying to control lateral balance (56). However, backward walking/stepping is essential for functional ambulation as it is required for many common activities of daily living like opening a door, stepping back from a curb to avoid a fast-moving vehicle etc. and backward balance loss often causes serious injuries as well (57) because backward falls are harder to prevent than forward and lateral ones (58). In fact, fallers show greater deficits in backward walking than non-fallers (59) and backward gait velocity identifies fallers more accurately than forward velocity (60). Backward walking has been applied effectively in several studies mostly with patient groups (61, 62).

Like during any motor learning task, multidirectional stepping causes structural and functional changes at the central nervous system. In order to achieve greater motor learning (in our case safer, smoother and more efficient multidirectional stepping performance) it is important to increase complexity and variability (63), which results in a flexible and adaptable motor system (63). In the StepIt exercise program this is achieved through the execution of steps in all directions and with different amplitudes, something which increases motor exploration and requires high inter- and intra-limb coordination, both of which are fundamental to human motor control, especially for foot trajectory and foot placement accuracy.

Finally, considering the limited generalization to other locomotor tasks (64) (stepping in different directions than the trained ones), falls prevention stepping training programs even for high-functioning older adults should incorporate multidirectional steps and also focus on the adaptation of step length (65) in order to be effective.

### Pattern Length

As mentioned earlier, in the StepIt exercise program, participants are presented with stepping patterns that they have to memorize and then execute on a grid-like mat. The number of steps inside the stepping patterns is progressively increased throughout the course of the intervention. Essentially, this is an n-back (1-back) visuospatial working memory task.

Working memory is fundamental to human cognition as it is responsible for maintaining and manipulating goal-relevant information for the performance of complex tasks (66). Working memory training has been shown to improve working memory capacity even in older adults, as a result of cognitive plasticity. Since cognitive plasticity starts when the environmental demands are higher than the demands the cognitive system usually faces (67), it is important that the difficulty level remains challenging (always a little higher than the routine performance level). Indeed, studies have demonstrated that training with variability in working memory task demands leads to greater transfer effects than training with constant task demands (68).

Although the exact procedures of how the neural systems that support working memory are altered through intensive training are not fully elucidated a recent neuroimaging study found that intensive working memory training produces functional changes in large-scale front parietal networks (69).

### Dual-/Multi-Tasking

Besides increasing the amount of step positions to be memorized, another way to increase cognitive load is to step while conducting additional motor or cognitive tasks.

It is well-documented that walking under cognitive load, a situation that is extremely common in everyday life, poses high motor and attentional demands on the central nervous system. Based on the capacity sharing theory, performing an additional task while walking alters gait performance and/or the execution of the secondary/cognitive task (70). Studies have extensively reported benefits of (esp. cognitive-motor) dual-task training on cognitive, motor (71) as well as dual-task performance (17, 72). Especially regarding dual-tasking and RAS, a process applied at the later stages of the StepIt exercise program, it is suggested that dual-tasking with rhythmic auditory cueing frees up cognitive resources (73).

Overall, a combination of physical exercise, sensorimotor stimulation and cognitive engagement may facilitate neurophysiological changes that contribute to cognitive improvement. A very recent review (74) has summarized the neurophysiological mechanisms underlying cognitive improvements following motor-cognitive dual-task training: dual-task training may stimulate similar neurobiological processes which produce a synergistic response: Common increase of cerebral blood flow as well as angiogenesis in the cortex and cerebellum induced from both physical exercise as well as cognitive training.

## Training Protocol

In this section, an exemplary protocol for 9 weeks of the group-based version of the StepIt exercise program aiming to improve physical, cognitive and psychological fall risk factors will be presented. The difficulty level described here is appropriate for older adults without major mobility or cognitive impairments, thus it can be used as a prevention (rather than rehabilitation) tool. Further recommendations regarding possible adjustments of this protocol to fit other populations, purposes and settings are presented in the “Design Recommendations” section.

### Session Structure and Equipment

**Figure 2:**
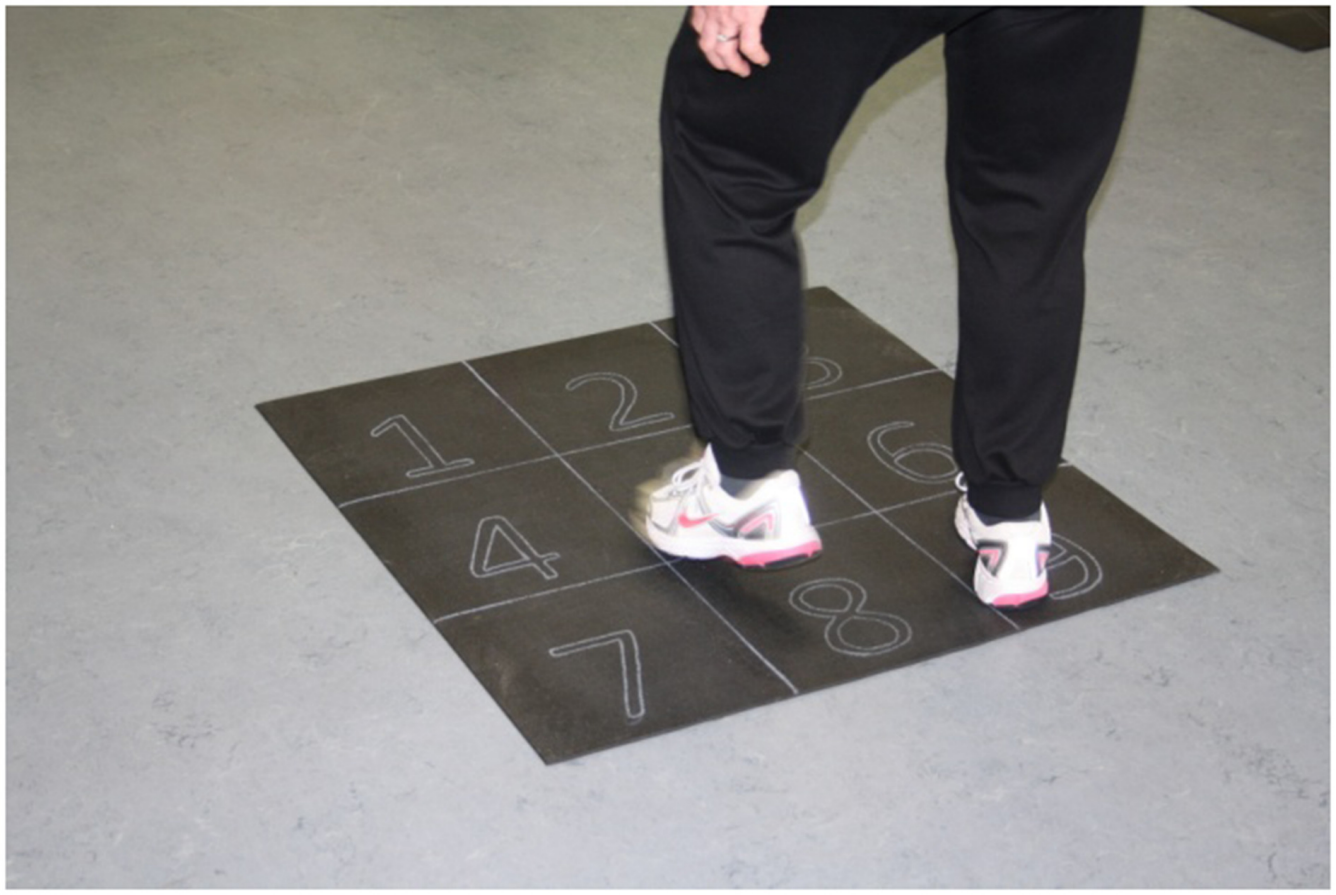
The StepIt mat.

The first 10 min of each session is a warm-up phase. First, participants can walk freely across the room at their own preferred speed while performing different joint-mobility exercises such as: arm circles forwards and backwards, walk on tip-toes, walk with butt kicks, walk with alternating knee lifting, side shuffle walking etc. Afterwards, rubber mats (one for each participant) are spread on the floor across the whole room. Their size should be approximately 90 × 90 cm and they should be made from extra non-slip yoga mats. They are divided into 9 equal squares (30 × 30 cm) with a 3 × 3 pattern (Figure 2). While participants are walking across the room, the instructor calls random numbers (from 1 to 9) and participants have to find the closest square on one of the mats with the number called by the instructor and step on it.

For the session's main phase (45 min) each participant stands behind their mat and starts performing the instructed stepping patterns. All sessions should be supervised by preferably two instructors. The main instructor (MI) leads the sessions and the assistant instructor (AI) stands at the back and offers help/correction when needed.

The MI demonstrates and explains the patterns first on a flipchart, which ensures good visibility also for participants standing at the back, and if needed also performs the pattern on his/her mat. Participants are then given time to practice the pattern until they can memorize and reproduce it without looking at the MI/flipchart. During this time both instructors walk around and provide help in case some participants need it. After a couple of rounds of practicing in their self-selected speed, a metronome is switched on and participants have to execute the stepping patterns in the given pace. Each session includes stepping patterns executed with one leg (left leg remained in the predefined start square and right leg stepped on the given numbered squares, then the same (i.e., mirrored) pattern was executed with the right leg remaining at the start square and the left stepped on the given numbered squares) as well as patterns with both legs moving in turns (to avoid confusion, execution should always with the same leg).

Moving to the new pattern happens once ca. 80% of the participants master the current pattern. In case some participants manage to learn the sequence much earlier than others, in order to avoid longer periods of inactivity and boredom, they can be approached from either the MI or the AI and asked to recall and repeat the patterns that have been taught so far in the session or to learn new patterns (with the same difficulty level) which the instructors come up with spontaneously.

Around 306 patterns should be developed for 18 training sessions. The number of patterns decreases every 2 weeks (because the number of steps within each pattern increases every 2 weeks) in order to have enough time to practice the patterns within the 45 min stepping phase. For a detailed description of the number of patterns for each condition and week, please see Table 1.

**Table 1:** Number of patterns to be practiced for each condition.

Week 1–3	6 patterns RF
	6 patterns LF
	8 patterns BF
Week 4–6	5 patterns RF
	5 patterns LF
	7 patterns BF
Week 7–9	4 patterns RF
	4 patterns LF
	6 patterns BF

*RF, right foot; LF, left foot; BF, both feet*.

In case this concept is used as an intervention with fixed duration, the exact content of each session (including the exact stepping patterns, the pace as well as the starting point/square for each of the 18 sessions) should be predefined and put together in a manual (see Supplementary Material for some exemplary training plans of week 1, 4, and 7) to be followed by all MIs.

The last 5 min of each session is a cool-down phase, where various stretching exercises in a standing position are performed.

### Progression

The difficulty level is gradually increased within each session (by using first one leg and then both for the execution of the stepping patterns) as well as between sessions.

Between sessions, the difficulty level is increased based on four principles (P); two of them addressing the increase of motor (M) load and two of them addressing the increase of cognitive (C) load (Table 2):

- Execution speed (PM1): starting from 92 beats per minute (BPM) for the single-leg and 64 BPM for both leg patterns to 118 and 104 BPM, respectively
- Direction and amplitude of the steps (pattern complexity) (PM2): starting from small forward and lateral steps to larger backward and oblique steps
- Number of steps for each pattern (pattern length) (PC1): starting from 3-step up to 8-step patterns
- Additional motor/cognitive tasks (Dual-/Multi-tasking) (PC2): starting with execution in Single-task condition (ST) to Dual- (DT) and then Multi-task (MT) condition.

**Table 2:** Difficulty level increase between sessions.

**Week**	**Steps**	**Pace (BPM)**		**Direction of feet placement**	**Additional task**
		**RF/LF**	**BF**		
1	3	92–100	64–78	Forward, side, and back	Single-task (ST)
2	4	100–108	78–86		
3		108–110	86–88		
4	5	110–112	88–90	Forward, side and back and skipping middle line (SML)	Dual-tasking (DT)
5	6	110	90–92		
6		112	94–96		
7	7	114	96–98	Forward, side, back, SML, and oblique steps	Multi-tasking (MT)
8	8	116	98–100		
9		118	102–104		

*BMP, beats per minute; RF, right foot; LF, left foot; BF, both feet*.

Regarding the direction of feet placement, for the first 3 weeks the patterns require forward, sideward and backward steps. In week 4–6 the patterns include much longer steps which require to skip the middle line of the squares (for example stepping from 7 to 2 etc.), and finally the last 3 weeks of the intervention, the patterns also require steps with crossings of the legs (for example from 6 to 5 or from 6 to 2).

Pacing of the steps is increased by 2 BPM at almost every session both for the RF/LF as well as the BF patterns.

Initially, the patterns include 3 steps and every 1 or 2 weeks the number of steps included in each pattern increases by 1 step, resulting in week 8–9 when patterns consist of 8 steps.

The last way to increase the difficulty level is the progress from single-task (only reproducing the stepping patterns) in week 1–3, to dual tasking in week 4–6, when participants will have to also conduct an additional motor (e.g., balance an object on their hands, clap hands front and behind their backs, browsing magazines, unbutton their shirt, hold ball above their heads/behind their backs etc.) or cognitive task (targeting two executive functions: verbal fluency (naming words associated with certain given words, naming objects, animals, vegetables, professions that start or end with a certain letter etc.) and memory/concentration (counting onwards, serial sevens, serial threes, spelling words backwards etc.) while executing the stepping pattern. Finally, in the last 3 weeks, while executing the stepping pattern, participants have to conduct both a motor and a cognitive task simultaneously (multi-tasking). Table 3 gives an overview of the content of all 18 training sessions.

**Table 3:** Detailed overview of the content of all 18 training sessions.

**Week**	**Session**	**Content**		**Session**	**Content**	
1	1	6 P:RF/LF and 8 P: BF	FSB	2	6 P:RF/LF and 8 P: BF	FSB
		92 BPM: RF/LF	3 Steps		100 BPM: RF/LF	3 Steps
		64 BPM: BF	Single-task		78 BPM: BF	Single task
2	3	6 P:RF/LF and 8 P: BF	FSB	4	6 P:RF/LF and 8 P: BF	FSB
		100 BPM: RF/LF	4 Steps		108 BPM: RF/LF	4 Steps
		78 BPM: BF	Single-task		86 BPM: BF	Single-task
3	5	6 P:RF/LF and 8 P: BF	FSB	6	6 P:RF/LF and 8 P: BF	FSB
		108 BPM: RF/LF	4 Steps		110 BPM: RF/LF	4 Steps
		86 BPM: BF	Single-task		88 BPM: BF	Single-task
4	7	5 P:RF/LF and 7 P: BF	FSB and SML	8	5 P:RF/LF and 7 P: BF	FSB and SML
		110 BPM: RF/LF	5 Steps		110 BPM: RF/LF	5 Steps
		88 BPM: BF	Dual-task		90 BPM: BF	Dual-task
5	9	5 P:RF/LF and 7 P: BF	FSB and SML	10	5 P:RF/LF and 7 P: BF	FSB and SML
		110 BPM: RF/LF	6 Steps		110 BPM: RF/LF	6 Steps
		90 BPM: BF	Dual-task		92 BPM: BF	Dual-task
6	11	5 P:RF/LF and 7 P: BF	FSB and SML	12	5 P:RF/LF and 7 P: BF	FSB and SML
		112 BPM: RF/LF	6 Steps		112 BPM: RF/LF	6 Steps
		94 BPM: BF	Dual-task		96 BPM: BF	Dual-task
7	13	4 P:RF/LF and 6 P: BF	FSB and SML and OS	14	4 P:RF/LF and 6 P: BF	FSB and SML and OS
		114 BPM: RF/LF	7 Steps		114 BPM: RF/LF	7 Steps
		96 BPM: BF	Multi-task		98 BPM: BF	Multi-task
8	15	4 P:RF/LF and 6 P: BF	FSB and SML and OS	16	4 P:RF/LF and 6 P: BF	FSB and SML and OS
		116 BPM: RF/LF	8 Steps		116 BPM: RF/LF	8 Steps
		98 BPM: BF	Multi-task		100 BPM: BF	Multi-task
9	17	4 P:RF/LF and 6 P: BF	FSB and SML and OS	18	4 P:RF/LF and 6 P: BF	FSB and SML and OS
		118 BPM: RF/LF	8 Steps		118 BPM: RF/LF	8 Steps
		102 BPM: BF	Multi-task		104 BPM: BF	Multi-task

*RF, right foot; LF, left foot; BF, both feet; BPM, beats per minute; P, patterns; FSB, forward, sideward (lateral), backward steps; SML, skipping middle line; OS, oblique steps*.

## Design recommendations

As mentioned earlier, the presented concept can be used as a framework for the development of further prevention and/or rehabilitation stepping exercise programs. In this section, we present recommendations on how this exercise program can be adjusted to fit different purposes, populations and settings. Being a form of highly specific balance training, this stepping training program can be applied to either healthy older adults aiming to improve their overall balance ability, as well as to neurological patients aiming to improve their stepping capacity and/or to any other fall-prone target group aiming to prevent falling.

It can be delivered either as a group-based training program, using mats and at least one instructor (possibly also two, depending on size and homogeneity of the group) or as a home-based training program using an ICT-based solution (e.g., in form of an exergame), which would not necessarily require the presence of an instructor. In clinical settings (e.g., physiotherapy practices or nursing home facilities), it is also possible to offer this mat-based but still in 1:1 training. Depending on the participants' preferences, RAS can be delivered via a metronome, music, or a combination of both. All of them have proven to be equally effective.

There are several ways to make training more variable or more challenging for example by altering material, position and size of the mat. Using a very thin mat (or even “drawing” the 9-square grid on the floor) is suitable for beginners or fall-prone older adults. At later stages or for relatively healthy older adults, thicker/softer mats can be used or the thin mats can be placed on unstable surfaces (e.g., on the Posturomed balance system), which will increase proprioception and neuromuscular demands.

In order to also train step length, the size of the mat can easily be personalized using the Pythagoras theorem by measuring maximum step length (MSL) with the maximum step length test (75) (or alternatively leg length or height). Beginners or fall-prone older adults can then start by training with mats sized to fit ca. 60% of their MSL and then progress up until 80% of their MSL. If personalizing mat size is not possible, it is possible to have three standard sizes (Small: 85 × 85 cm, Normal: 90 × 90 cm and Big: 95 × 95 cm) and use them based on participant's height and/or training level.

Regarding tempo and dosage, a recent meta-analysis on the effects of RAS in Parkinson's patients (24) found that training using this method should include tempo variations ±10% with respect to the preferred cadence, for a minimal period of 20–45 min per day, for at least 3–5 days per week. Although these recommendations cannot be used as is for a stepping training because they are based on gait studies, they can be used as a basis and adjusted to fit needs and fitness levels of other kinds of neurological patients (stroke, MS) as well as healthy older adults.

Furthermore, content/focus of training can also be adjusted to fit needs of different target groups. Participants with memory complaints or mild cognitive impairment can focus on the cognitive load (pattern length and dual-/multi-tasking) whereas stroke/Parkinson's patients can focus on the motor load (execution speed and pattern complexity).

## Conclusions

To reduce the burden of falls in older adults falls prevention exercise programs that apply new research evidence into practice need to be developed. The ability to execute movements varying in speed, amplitude, complexity and additional cognitive load is critical for preventing falls (76) and thus stepping training programs that incorporate such aspects have resulted in substantial reductions of falls (15).

We have proposed a framework for a stepping training program, reviewed the theory and evidence underlying it and described in detail the implementation of an exemplary 9 week, group-based stepping exercise program applying the suggested concept.

The exemplary training plan does not require costly equipment and has potential for high adherence levels, taking into account the social aspect of physical activity.

However, being a group-based, it also has certain limitations such as that, the use of relevant secondary cognitive tasks (e.g., visual search tasks) is not possible. Moreover, adjustments of the difficulty level cannot happen according to each participant's current personal level. However, recent evidence suggests that even just exposure to varying levels of task difficulty is sufficient for inducing training gains (77), making individually-tailored training not always necessary.

The elements as well as progression rate of the exemplary training program can be easily modified to fit the needs of different samples (e.g., healthy older adults, patient groups), which makes it a useful tool for the development of further stepping exercise programs. Pilot feasibility studies are needed to test its feasibility (safety/adverse outcomes and enjoyment/adherence). If proven feasible, its effectiveness to improve fall risk factors is then to be tested via randomized controlled trials. Due to its multi-domain approach, besides the improvement of stepping capacity, further expected outcomes include the improvement of further physical, cognitive and psychological risk factors. Thus, further studies using this exercise regimen or modified versions of it are encouraged.

## Data Availability Statement

All data for this study have been provided in the article/Supplementary Material.

## Author Contributions

EG developed the original research idea, designed the study, and was the major contributor in writing this manuscript. TM and WZ contributed significant components to the study design. All authors critically revised and approved the manuscript.

### Conflict of Interest

The authors declare that the research was conducted in the absence of any commercial or financial relationships that could be construed as a potential conflict of interest.

## References

[1] Rubenstein LZ Josephson KR. The epidemiology of falls and syncope. Clin Geriatr Med. (2002) 18:141–58. 10.1016/S0749-0690(02)00002-2 12180240

[2] Chapman GJ Hollands MA. Evidence for a link between changes to gaze behaviour and risk of falling in older adults during adaptive locomotion. Gait Posture. (2006) 24:288–94. 10.1016/j.gaitpost.2005.10.002 16289922

[3] Schoene D Delbaere K Lord SR. Impaired response selection during stepping predicts falls in older people—a cohort study. J Am Med Dir Assoc. (2017) 18:719–25. 10.1016/j.jamda.2017.03.010 28526585

[4] Melzer I Kurz I Shahar D Oddsson LI. Do voluntary step reactions in dual task conditions have an added value over single task for fall prediction? A Prospective Study. Aging Clin Exp Res. (2010) 22:6–360. 10.1007/BF03324940 21422793

[5] Chapman GJ Hollands MA. Evidence that older adult fallers prioritise the planning of future stepping actions over the accurate execution of ongoing steps during complex locomotor tasks. Gait Posture. (2007) 26:59–67. 10.1016/j.gaitpost.2006.07.010 16939711

[6] Chapman GJ Hollands MA. Age-related differences in stepping performance during step cycle-related removal of vision. Exp Brain Res. (2006) 174:613–21. 10.1007/s00221-006-0507-6 16733708

[7] Maki BE Edmondstone MA McIlroy WE. Age-related differences in laterally directed compensatory stepping behavior. J Gerontol Ser A. (2000) 55:M270–7. 10.1093/gerona/55.5.M270 10819317

[8] Mille ML Johnson-Hilliard M Martinez KM Zhang Y Edwards BJ Rogers MW. One step, two steps, three steps more. Directional vulnerability to falls in community-dwelling older people. J Gerontol Ser A. (2013) 68:1540–8. 10.1093/gerona/glt062 23685768 PMC3814241

[9] Pijnappels M Delbaere K Sturnieks DL Lord SR. The association between choice stepping reaction time and falls in older adults–a path analysis model. Age Ageing. (2010) 39:99–104. 10.1093/ageing/afp200 20015855

[10] Verhaeghen P Steitz DW Sliwinski MJ Cerella J. Aging and dual-task performance: a meta-analysis. Psychol Aging. (2003) 18:443–60. 10.1037/0882-7974.18.3.443 14518807

[11] Sherrington C Fairhall NJ Wallbank GK Tiedemann A Michaleff ZA Howard K Exercise for preventing falls in older people living in the community. Cochrane Database Syst Rev. (2019) CD012424. 10.1002/14651858.CD012424.pub2 PMC6360922 30703272

[12] Luchies CW Alexander NB Schultz AB Ashton-Miller J. Stepping responses of young and old adults to postural disturbances: kinematics. J Am Geriatr Soc. (1994) 42:506–12. 10.1111/j.1532-5415.1994.tb04972.x 8176145

[13] Lord SR Fitzpatrick RC. Choice stepping reaction time: a composite measure of falls risk in older people. J Gerontol Ser A. (2001) 56:M627–32. 10.1093/gerona/56.10.M627 11584035

[14] Maki BE McIlroy WE. Control of rapid limb movements for balance recovery: age-related changes and implications for fall prevention. Age Ageing. (2006) 35(Suppl. 2):ii12–18. 10.1093/ageing/afl078 16926197

[15] Okubo Y Schoene D Lord SR. Step training improves reaction time, gait and balance and reduces falls in older people: a systematic review and meta-analysis. Br J Sports Med. (2016) 51:586–93. 10.1136/bjsports-2015-095452 26746905

[16] Liu-Ambrose T Best JR Exercise is medicine for the aging brain. Kinesiol. Rev. (2017) 6:22–9. 10.1123/kr.2016-0035

[17] Wollesen B Voelcker-Rehage C Training effects on motor–cognitive dual-task performance in older adults. Eur Rev Aging Phys Activ. (2014) 11:5–24. 10.1007/s11556-013-0122-z

[18] Sherrington C Michaleff ZA Fairhall N Paul SS Tiedemann A Whitney J. Exercise to prevent falls in older adults: an updated systematic review and meta-analysis. Br J Sports Med. (2016) 51:1750–8. 10.1136/bjsports-2016-096547 27707740

[19] Skjæret-Maroni N Vonstad EK Ihlen EA Tan XC Helbostad JL Vereijken B. Exergaming in older adults: movement characteristics while playing stepping games. Front Psychol. (2016) 7:964. 10.3389/fpsyg.2016.00964 27445926 PMC4919354

[20] Thaut M Rhythm, Music, and the Brain: Scientific Foundations and Clinical Applications. (2013). Available online at: https://books.google.de/books?hl=deandlr=andid=j25HB8yND80Candoi=fndandpg=PP2anddq=thaut+2005+rhythmicandots=LR47egmuYiandsig=Uo20AON3zzPPZ388BYYuh7Os1a4#v=onepageandq=thaut2005rhythmicandf=false (assessed March 16, 2018).

[21] Thaut MH Abiru M Rhythmic auditory stimulation in rehabilitation of movement disorders: a review of current research. Music Percept. (2010) 27:263–9. 10.1525/mp.2010.27.4.263

[22] Wright RL Bevins JW Pratt D Sackley CM Wing AM. Metronome cueing of walking reduces gait variability after a cerebellar stroke. Front Neurol. (2016) 7:84. 10.3389/fneur.2016.00084 27313563 PMC4887482

[23] Yoo GE Kim SJ. Rhythmic auditory cueing in motor rehabilitation for stroke patients: systematic review and meta-analysis. J Music Ther. (2016) 53:149–77. 10.1093/jmt/thw003 27084833

[24] Ghai S Ghai I Schmitz G Effenberg AO. Effect of rhythmic auditory cueing on Parkinsonian gait: a systematic review and meta-analysis. Sci Rep. (2018) 8:506. 10.1038/s41598-017-16232-5 29323122 PMC5764963

[25] Spaulding SJ Barber B Colby M Cormack B Mick T Jenkins ME. Cueing and gait improvement among people with parkinson's disease: a meta-analysis. Arch Phys Med Rehabil. (2013) 94:562–70. 10.1016/j.apmr.2012.10.026 23127307

[26] Shahraki M Sohrabi M Taheri Torbati HR Nikkhah K NaeimiKia M. Effect of rhythmic auditory stimulation on gait kinematic parameters of patients with multiple sclerosis. J Med Life. (2017) 10:33–7. 28255373 PMC5304368

[27] Wright RL Brownless SB Pratt D Sackley CM Wing AM. Stepping to the beat: feasibility and potential efficacy of a home-based auditory-cued step training program in chronic stroke. Front Neurol. (2017) 8:412. 10.3389/fneur.2017.00412 28878730 PMC5572237

[28] Wright RL Masood A MacCormac E Pratt D Sackley CM Wing AM Metronome-cued stepping in place after hemiparetic stroke: comparison of a one- and two-tone beat. ISRN Rehabil. (2013) 2013:1–5. 10.1155/2013/157410

[29] Thaut MH McIntosh GC Rice RR. Rhythmic facilitation of gait training in hemiparetic stroke rehabilitation. J Neurol Sci. (1997) 151:207–12. 10.1016/S0022-510X(97)00146-9 9349677

[30] Hausdorff JM Lowenthal J Herman T Gruendlinger L Peretz C Giladi N. Rhythmic auditory stimulation modulates gait variability in Parkinson's disease. Eur J Neurosci. (2007) 26:2369–75. 10.1111/j.1460-9568.2007.05810.x 17953624

[31] Grahn JA Brett M. Rhythm and beat perception in motor areas of the brain. J Cogn Neurosci. (2007) 19:893–906. 10.1162/jocn.2007.19.5.893 17488212

[32] Shelton J Kumar PG Comparison between auditory and visual simple reaction times. Neurosci Med. (2010) 1:30–2. 10.4236/nm.2010.11004

[33] Thaut MH Kenyon GP. Rapid motor adaptations to subliminal frequency shifts during syncopated rhythmic sensorimotor synchronization. Hum Mov Sci. (2003) 22:321–38. 10.1016/S0167-9457(03)00048-4 12967761

[34] Thaut MH McIntosh GC Hoemberg V. Neurobiological foundations of neurologic music therapy: rhythmic entrainment and the motor system. Front Psychol. (2015) 5:1185. 10.3389/fpsyg.2014.01185 25774137 PMC4344110

[35] Thaut MH Kenyon GP Schauer ML McIntosh GC. The connection between rhythmicity and brain function. IEEE Eng. Med. Biol. Mag. (1999) 18:101–8. 10.1109/51.752991 10101675

[36] Nombela C Hughes LE Owen AM Grahn JA. Into the groove: can rhythm influence parkinson's disease?” Neurosci Biobehav Rev. 37, 2564–2570. 10.1016/j.neubiorev.2013.08.003 24012774

[37] Repp BH. Sensorimotor synchronization and perception of timing: effects of music training and task experience. Hum Mov Sci. (2010) 29:200–13. 10.1016/j.humov.2009.08.002 20074825

[38] Ghai S Ghai I Effenberg AO. Effect of rhythmic auditory cueing on aging gait: a systematic review and meta-analysis. Aging Dis. (2018) 9:901–23. 10.14336/AD.2017.1031 30271666 PMC6147584

[39] Schmidt RA Lee TD Motor Control and Learning : A Behavioral Emphasis. Human Kinetics. Champaign, IL (1999).

[40] Hinkel-Lipsker JW Hahn ME. The effects of variable practice on locomotor adaptation to a novel asymmetric gait. Exp Brain Res. (2017) 235:2829–41. 10.1007/s00221-017-5015-3 28647814

[41] Granacher U Muehlbauer T Zahner L Gollhofer A Kressig RW. Comparison of traditional and recent approaches in the promotion of balance and strength in older adults. Sports Med. (2011) 41:377–400. 10.2165/11539920-000000000-00000 21510715

[42] van den Bogert AJ Pavol MJ Grabiner MD. Response time is more important than walking speed for the ability of older adults to avoid a fall after a trip. J Biomech. (2002) 35:199–205. 10.1016/S0021-9290(01)00198-1 11784538

[43] Danilovich MK Conroy DE Hornby TG. Feasibility and impact of high-intensity walking training in frail older adults. J Aging Phys Activ. (2017) 25:533–8. 10.1123/japa.2016-0305 28120633

[44] Sullivan KJ Knowlton BJ Dobkin BH. Step training with body weight support: effect of treadmill speed and practice paradigms on poststroke locomotor recovery. Arch Phys Med Rehabil. (2002) 83:683–91. 10.1053/apmr.2002.32488 11994808

[45] Vasudevan EV Bastian AJ. Split-belt treadmill adaptation shows different functional networks for fast and slow human walking. J Neurophysiol. (2010) 103:183–91. 10.1152/jn.00501.2009 19889853 PMC2807217

[46] Yokoyama H Ogawa T Kawashima N Shinya M Nakazawa K. Distinct sets of locomotor modules control the speed and modes of human locomotion. Sci Rep. (2016) 6:36275. 10.1038/srep36275 27805015 PMC5090253

[47] Patla AE Adkin A Ballard T. Online steering: coordination and control of body center of mass, head and body reorientation. Exp Brain Res. (1999) 129:0629–34. 10.1007/s002210050932 10638436

[48] Berg WP Alessio HM Mills EM Tong C. Circumstances and consequences of falls in independent community-dwelling older adults. Age Ageing. (1997) 26:261–8. 10.1093/ageing/26.4.261 9271288

[49] Hill K Schwarz J Flicker L Carroll S. Falls among healthy, community-dwelling, older women: a prospective study of frequency, circumstances, consequences and prediction accuracy. Aust N Z J Public Health. (1999) 23:41–8. 10.1111/j.1467-842X.1999.tb01203.x 10083688

[50] Crenshaw JR Bernhardt KA Achenbach SJ Atkinson EJ Khosla S Kaufman KR. The circumstances, orientations, and impact locations of falls in community-dwelling older women. Arch Gerontol Geriatr. (2017) 73:240–7. 10.1016/j.archger.2017.07.011 28863352 PMC5858880

[51] Pai YC Rogers MW Patton J Cain TD Hanke TA. Static versus dynamic predictions of protective stepping following waist–pull perturbations in young and older adults. J Biomech. (1998) 31:1111–8. 10.1016/S0021-9290(98)00124-9 9882043

[52] Patla AE Frank JS Winter DA Rietdyk S Prentice S Prasad S. Age-related changes in balance control system: initiation of stepping. Clin Biomech. (1993) 8:179–84. 10.1016/0268-0033(93)90012-7 23915967

[53] Medell JL Alexander NB. A clinical measure of maximal and rapid stepping in older women. J Gerontol Med Sci Public Domain. (2000) 55:M429–33. 10.1093/gerona/55.8.M429 10952364

[54] Robinovitch SN Feldman F Yang Y Schonnop R Leung PM Sarraf T. Video capture of the circumstances of falls in elderly people residing in long-term care: an observational study. Lancet. (2013) 381:47–54. 10.1016/S0140-6736(12)61263-X 23083889 PMC3540102

[55] Greenspan SL Myers ER Kiel DP Parker RA Hayes WC Resnick NM. Fall direction, bone mineral density, and function: risk factors for hip fracture in frail nursing home elderly. Am J Med. (1998) 104:539–45. 10.1016/S0002-9343(98)00115-6 9674716

[56] Mille ML Johnson ME Martinez KM Rogers MW. Age-dependent differences in lateral balance recovery through protective stepping. Clin Biomech. (2005) 20:607–16. 10.1016/j.clinbiomech.2005.03.004 15890438

[57] Kadono N Pavol MJ. Effects of aging-related losses in strength on the ability to recover from a backward balance loss. J Biomech. (2013) 46:13–8. 10.1016/j.jbiomech.2012.08.046 23146321

[58] Hsiao ET Robinovitch SN. Common protective movements govern unexpected falls from standing height. J Biomech. (1997) 31:1–9. 10.1016/S0021-9290(97)00114-0 9596532

[59] Fritz NE Worstell AM Kloos AD Siles AB White SE Kegelmeyer DA. Backward walking measures are sensitive to age-related changes in mobility and balance. Gait Posture. (2013) 37:593–7. 10.1016/j.gaitpost.2012.09.022 23122938

[60] Kurup HV Clark CI Dega RK. Footwear and orthopaedics. Foot Ankle Surg. (2012) 18:79–83. 10.1016/j.fas.2011.03.012 22443991

[61] Boone T Astorino TA Baker JS Brock S Dalleck LC Goulet EDB Backward walking: a possible active exercise for low back pain reduction and enhanced function in athletes. J Exerc Physiol. (2011) 14:17–26.

[62] Yang YR Yen JG Wang RY Yen LL Lieu FK. Gait outcomes after additional backward walking training in patients with stroke: a randomized controlled trial. Clin Rehabil. (2005) 19:264–73. 10.1191/0269215505cr860oa 15859527

[63] Wu HG Miyamoto YR Gonzalez Castro LN Ölveczky BP Smith MA. Temporal structure of motor variability is dynamically regulated and predicts motor learning ability. Nat Neurosci. (2014) 17:312–21. 10.1038/nn.3616 24413700 PMC4442489

[64] Choi JT Bastian AJ. Adaptation reveals independent control networks for human walking. Nat Neurosci. (2007) 10:1055–62. 10.1038/nn1930 17603479

[65] Hak L Houdijk H Beek PJ van Dieën JH. Steps to take to enhance gait stability: the effect of stride frequency, stride length, and walking speed on local dynamic stability and margins of stability. PLoS ONE. (2013) 8:e82842. 10.1371/journal.pone.0082842 24349379 PMC3862734

[66] Baddeley A. Working Memory. Science. (1992) 255:556–9. 10.1126/science.1736359 1736359

[67] Lövdén M Bäckman L Lindenberger U Schaefer S Schmiedek F. A theoretical framework for the study of adult cognitive plasticity. Psychol Bull. (2010) 136:659–76. 10.1037/a0020080 20565172

[68] Schmidt RA Bjork RA New conceptualizations of practice: common principles in three paradigms suggest new concepts for training. Psychol Sci. (1992) 3:207–18. 10.1111/j.1467-9280.1992.tb00029.x

[69] Thompson TW Waskom ML Gabrieli JD. Intensive working memory training produces functional changes in large-scale frontoparietal networks. J Cogn Neurosci. (2016) 28:575–88. 10.1162/jocn_a_00916 26741799 PMC5724764

[70] Friedman A Polson MC Dafoe CG Gaskill SJ. Dividing attention within and between hemispheres: testing a multiple resources approach to limited-capacity information processing. J Exp Psychol Hum Percept Perform. (1982) 8:625–50. 10.1037/0096-1523.8.5.625 6218226

[71] Ghai S Ghai I Effenberg AO. Effects of dual tasks and dual-task training on postural stability: a systematic review and meta-analysis. Clin Interv Aging. (2017) 12:557–77. 10.2147/CIA.S125201 28356727 PMC5367902

[72] Agmon M Belza B Nguyen HQ Logsdon RG Kelly VE. A systematic review of interventions conducted in clinical or community settings to improve dual-task postural control in older adults. Clin Interv Aging. (2014) 9:477–92. 10.2147/CIA.S54978 24741296 PMC3970921

[73] Lohnes CA Earhart GM. The impact of attentional, auditory, and combined cues on walking during single and cognitive dual tasks in Parkinson disease. Gait Posture. (2011) 33:478–83. 10.1016/j.gaitpost.2010.12.029 21273075

[74] Tait JL Duckham RL Milte CM Main LC Daly RM. Influence of sequential vs. simultaneous dual-task exercise training on cognitive function in older adults. Front Aging Neurosci. (2017) 9:368. 10.3389/fnagi.2017.00368 29163146 PMC5681915

[75] Goldberg A Schepens S Wallace M. Concurrent validity and reliability of the maximum step length test in older adults. J Geriatr Phys Ther. (2010) 33:122–7. 10.1097/JPT.0b013e3181eda302 21155507

[76] Grabiner MD Crenshaw JR Hurt CP Rosenblatt NJ Troy KL. Exercise-based fall prevention: can you be a bit more specific?” Exerc Sport Sci Rev. (2014) 42:161–8. 10.1249/JES.0000000000000023 25062002

[77] von Bastian CC Eschen A. Does working memory training have to be adaptive?” Psychol Res. (2016) 80:181–94. 10.1007/s00426-015-0655-z 25716189

