# Supplementary material for: A Novel Square-Stepping Exercise Program for Older Adults (StepIt): Rationale and Implications for Falls Prevention
Source: Front Med (Lausanne). 2020 Jan 14;6:318. doi: 10.3389/fmed.2019.00318 (PMC6970979; doi:10.3389/fmed.2019.00318)
Supplement: Supplementary file 1 [file Data_Sheet_1.pdf]

Supplementary Material

A novel square-stepping exercise program for older adults (StepIt); rationale and implications for falls prevention

Eleftheria Giannouli*, Tobias Morat, Wiebren Zijlstra

Institute of Movement and Sport Gerontology, German Sport University Cologne, Cologne, Germany

*** Correspondence:** Eleftheria Giannouli: eleftheria.giannouli@unibas.ch

Supplementary Figure 1: Exemplary extract of the StepIt trainers' manual for week 1

Right Foot Left Foot Both

Week 1	Session	Patterns (RF)	Patterns (LF)	Patterns (BF)
**3 Steps**<br><br>**Single-Task**<br><br>**Front Side Back Side (FBS)**<br><br>**Start with both feet @5**	1<br><br>**BPM**<br>**R&F: 96**<br><br>**BF: 84**	1. 2-3-6<br>2. 2-6-9<br>3. 8-3-6<br>4. 8-6-2<br>5. 3-5-9<br>6. 3-2-6	1. 2-1-4<br>2. 2-4-7<br>3. 8-1-4<br>4. 8-4-2<br>5. 1-5-7<br>6. 1-2-4	1. 6-4-3<br>2. 6-8-2<br>3. 2-1-6<br>4. 2-4-6<br>5. 3-2-6<br>6. 3-1-2<br>7. 9-7-8<br>8. 8-4-6
	2<br><br>**BPM**<br>**R&F: 98**<br><br>**BF: 88**	1. 2-8-6<br>2. 2-3-9<br>3. 6-2-5<br>4. 6-8-9<br>5. 3-9-6<br>6. 3-8-6	1. 2-8-4<br>2. 2-1-7<br>3. 4-2-5<br>4. 4-8-7<br>5. 1-7-4<br>6. 1-8-4	1. 6-1-3<br>2. 6-8-9<br>3. 9-8-6<br>4. 9-4-3<br>5. 8-7-6<br>6. 8-4-9<br>7. 2-4-8<br>8. 3-4-9

Note: RF: right foot, LF: left foot, BF: both feet, FSB: forward, sideward (lateral), BMP: beats per minute

Supplementary Figure 2: Exemplary extract of the StepIt trainers' manual for week 4

Right Foot Left Foot Both

Week 4	Session	Patterns (RF)	Patterns (LF)	Patterns (BF)
**5 Steps**<br><br>**Dual Task**<br><br>**FSB + Skipping Middle Line (SML)**<br><br>**Start with both feet @8**	7<br><br>**BPM**<br>**R&F: 102**<br><br>**BF: 92**	1. 5-3-6-9-2<br>2. 5-6-9-2-3<br>3. 2-3-6-5-2<br>4. 2-6-5-6-3<br>5. 3-5-9-2-5<br>_________________________<br>Cognitive: When I raise my right hand, you say "Red" when I raise my left arm you say "Green"	1. 5-1-4-7-2<br>2. 5-4-7-2-1<br>3. 2-1-4-5-2<br>4. 2-4-5-4-1<br>5. 1-5-7-2-5<br>_________________________<br>Cognitive: Whisper a story while you execute the stepping patterns	1. 6-4-2-7-9<br>2. 6-7-2-4-9<br>3. 2-1-4-7-3<br>4. 2-4-6-7-2<br>5. 3-5-6-1-5<br>6. 3-1-2-7-5<br>7. 9-5-3-2-9<br>_________________________<br>Motor1: Clap your hands in front of you and behind your back in turns<br>Motor2: Balance your cell phone on your hand
	8<br><br>**BPM**<br>**R&F: 104**<br><br>**BF: 94**	1. 3-2-8-9-3<br>2. 2-8-6-5-3<br>3. 2-3-9-3-5<br>4. 6-2-5-3-9<br>5. 9-3-9-2-8<br>_________________________<br>Motor: Stretch your arms to the top and to the side in turns	1. 1-2-8-7-1<br>2. 2-8-4-5-1<br>3. 2-1-7-1-5<br>4. 4-2-5-1-8<br>5. 7-1-7-2-8<br>_________________________<br>Motor: Fold and unfold a piece of paper	1. 6-1-3-7-9<br>2. 6-5-9-1-3<br>3. 9-5-3-1-2<br>4. 9-5-3-2-9<br>5. 5-7-2-1-3<br>6. 5-7-6-1-9<br>7. 6-7-2-4-9<br>_________________________<br>Cognitive1: Say as many words as you can associated with "summer"<br>Cognitive2: Say as many words as you can that begin with the letter "S"

Note: RF: right foot, LF: left foot, BF: both feet, FSB: forward, sideward (lateral), BMP: beats per minute

Supplementary Figure 3: Exemplary extract of the StepIt trainers' manual for week 7

		Right Foot	Left Foot	Both
**Week 7**	**Session**	**Patterns (RF)**	**Patterns (LF)**	**Patterns (BF)**
**7 Steps**<br><br>**Multi Task**<br><br>**FSB + SML + CROSS**<br><br>**Start with both feet @5**	13<br><br>**<u>BPM</u>**<br>**R&F: 114**<br><br>**BF: 96**	1. 2-3-6-7-9-5-8<br>2. 2-6-9-1-5-2-3<br>3. 8-3-6-2-8-1-9<br>4. 8-6-2-1-3-9-5<br>__________<br>When I raise my right hand, you say "STOP" and when I raise my left arm you say "GO" while you clap your hands in front of you and behind your back in turns	1. 2-1-4-9-7-5-8<br>2. 2-4-7-3-5-2-1<br>3. 8-1-4-2-8-3-7<br>4. 8-4-2-3-1-7-5<br>__________<br>Stretch your arms to the top and to the side in turns while you say as many words are you can associated with "winter"	1. 6-4-3-9-1-7-6<br>2. 6-8-5-2-7-4-9<br>3. 2-6-3-7-8-6-3<br>4. 2-9-6-4-5-9-6<br>5. 3-6-2-5-1-7-9<br>6. 3-1-2-9-5-3-6<br>__________<br>Multitask1: Whisper a story while you balance a pen on your hand<br><br>Multitask2: Stretch your arms to the top and to the side in turns while you say as many words as you can that begin with the letter "B"
	14<br><br>**<u>BPM</u>**<br>**R&F: 114**<br><br>**BF: 98**	1. 2-8-6-3-5-7-9<br>2. 2-3-9-5-1-2-8<br>3. 6-2-5-7-9-1-3<br>4. 6-8-9-1-3-7-5<br>__________<br>Stretch your arms to the top and to the side in turns while you say as many words are you can associated with "winter"	1. 2-8-4-1-5-9-7<br>2. 2-1-7-5-3-2-8<br>3. 4-2-5-9-7-3-1<br>4. 4-8-7-3-1-9-5<br>__________<br>When I raise my right hand, you say "GO" when I raise my left arm you say "STOP" while you clap your hands in front of you and behind your back in turns	1. 1-4-3-9-2-8-3<br>2. 8-6-9-3-8-1-2<br>3. 9-8-4-7-8-6-3<br>4. 9-2-7-1-6-8-2<br>5. 8-6-2-8-9-6-3<br>6. 8-4-1-9-5-2-6<br>__________<br>Multitask1: Whisper a story while you balance a pen on your hand<br>Multitask2: Stretch your arms to the top and to the side in turns while you say as many words as you can that begin with the letter "B"

Note: RF: right foot, LF: left foot, BF: both feet, FSB: forward, sideward (lateral), BMP: beats per minute
